# Correction: Effects of temperature on the life history traits of intermediate host snails of fascioliasis: A systematic review

**DOI:** 10.1371/journal.pntd.0014064

**Published:** 2026-03-05

**Authors:** Agrippa Dube, Chester Kalinda, Tawanda Manyangadze, Tafadzwa Mindu, Moses John Chimbari

The Funding statement for this article is incorrect. The correct Funding statement is as follows: This project has received funding from the European Union’s Horizon 2020 research and innovation programme under grant agreement no. 101000365.
