# Supplementary material for: Effects of temperature on the life history traits of intermediate host snails of fascioliasis: A systematic review
Source: PLoS Negl Trop Dis. 2023 Dec 4;17(12):e0011812. doi: 10.1371/journal.pntd.0011812 (PMC10721167; doi:10.1371/journal.pntd.0011812)
Supplement: Attachment — Submitted filename: Review.docx [file pntd.0011812.s001.docx]

Title: "Effects of Temperature on the Life History Traits of Intermediate Host Snails of Fascioliasis: A Systematic Review"

Introduction:

The paper explores the impact of temperature on intermediate host snails of fascioliasis. The background information provided effectively conveys the significance of the research topic, and the research question and objectives are clearly articulated. However, I would appreciate further elucidation of how the authors intend to support the hypothesis regarding temperature changes potentially being involved in triclabendazole resistance.

Materials and Methods:

The search strategy, inclusion and exclusion criteria are well-detailed.

Results:

The results are presented clearly, and appropriate criteria are employed to substantiate the findings. However, before proceeding with the review, I have some key questions that should be addressed:

1. The initial part of the results section appears to stem from the research strategy. It would be more logical to relocate this to the Materials and Methods section, thereby allowing the results section to focus exclusively on the biological effects of temperatures on the life history of the intermediate host of fasciolosis.

2. There is inconsistency in the formatting of species names, with some in italics and others not. Please ensure uniformity.

3. Please review the formatting of the "Continent" and "Temperature" title names in Table 1.

4. In Table 2, consider adding a range in the fourth column for completeness.

5. Although Radix sp has been cited in Table 1 and in the text, it is not clear how this data is related in both tables.

6. To enhance reader comprehension, the authors should avoid inserting paper codes in the middle of sentences.

7. An English review is necessary to refine the manuscript's elegance.

8. The inclusion of the mathematical modeling section in the results part requires clarification. It would be helpful to mention this in the Materials and Methods section and provide a summary of the most relevant models and outcomes.

Overall, this is a well-conducted study that provides valuable insights into temperature-dependent stages and the biology of IH snails and Fasciola spp. However, the results section lacks summarization of each presented topic. Moreover, the discussion section is challenging to follow, requiring a complete reorganization to clarify the influences of temperature ranges on IH snail biology (linking aspects such as survival, life history, growth, and reproduction). I recommend addressing these issues before proceeding with further consideration for publication.
