# Supplementary material for: Effects of temperature on the life history traits of intermediate host snails of fascioliasis: A systematic review
Source: PLoS Negl Trop Dis. 2023 Dec 4;17(12):e0011812. doi: 10.1371/journal.pntd.0011812 (PMC10721167; doi:10.1371/journal.pntd.0011812)
Supplement: Attachment — Submitted filename: Rebuttal letter Addressing Reviewers comments .docx [file pntd.0011812.s002.docx]

Agrippa Dube. Msc.

School of Nursing and Public Health

College of Health Sciences

Howard College Campus

University of KwaZulu-Natal

Durban 4001, South Africa

[adube168@gmail.com](mailto:adube168@gmail.com)

16/11/2023

The Editor-in-Chief

PLoS NTDs

Dear Sir/Madam,

**Ref**: Effect of temperature on life history traits of intermediate host snails of fascioliasis (Manuscript Number: PNTD-D-23-00997).

We acknowledge receipt of the reviews’ comments for the manuscript that was submitted to your journal for publication. Kindly find below a point-by-point rebuttal of the comments that were addressed.

Best,

Agrippa Dube.

| Line No | | Reviewers' comments | Author's response | | | Line edited | |
| --- | --- | --- | --- | --- | --- | --- | --- |
| **Reviewer 1** | | | | | | | |
| **Background** | | | | | | | |
|  | | The paper explores the impact of temperature on intermediate host snails of fascioliasis. The background information provided effectively conveys the significance of the research topic, and the research question and objectives are clearly articulated. However, I would appreciate further elucidation of how the authors intend to support the hypothesis regarding temperature changes potentially being involved in triclabendazole resistance. | **We have addressed the query in the revised manuscript. (In literature it was observed that climate change may produce favourable conditions for the outbreak and transmission of fascioliasis, and this might result in the repeated use of the current drug of choice triclabendazole against fascioliasis in endemic or new areas of the disease, resulting in mutant strains of flukes, which resist triclabendazole effects)[1]. This was the support in Literature: Fasciolosis-an increasing challenge in the sheep industry. Animals. 2022;12(12):1491*.*** | | | Lines 103-105 | |
| **Results** | | | | | | | |
|  | | The initial part of the results section appears to stem from the research strategy. It would be more logical to relocate this to the Materials and Methods section, thereby allowing the results section to focus exclusively on the biological effects of temperatures on the life history of the intermediate host of fasciolosis. | **In the revised manuscript we moved the initial part of the results to the materials and methods section as suggested: “ The systematic literature search yielded a total of one thousand two hundred twenty-seven plus seventeen reports from other sources, which included abstracts, reports, books, and theses (Figure 1). Seven hundred fifty articles were eliminated because of duplication, and three hundred articles were removed as they were deemed ineligible based on their titles and abstracts. A further one hundred and twenty articles were excluded because they did not focus on temperature despite looking at the life history traits of IHS, and thirty-nine were further dropped as they did not specifically report on temperature about the IHS *Lymnaea* spp, *Fossaria* spp, *Galba* spp, *Austropeplea* spp *Pseudosuccinea* spp, and *Radix* spp of *Fasciola* spp. Table 1 lists the thirty-five publications that were considered for this evaluation. The findings from the publications that met the criteria for the review were categorized into the following themes: temperature and fecundity, temperature and growth, temperature and survival, and temperature and parasite development. These are discussed below about potential changes in the disease risk with a possible rise in temperature”** | | | Lines135-146. | |
|  | | There is inconsistency in the formatting of species names, with some in italics and others not. Please ensure uniformity | **The issue of formatting parasite and snail species names was addressed in the revised manuscript.** | | |  | |
|  | | Please review the formatting of the "Continent" and "Temperature" title names in Table 1 | **The title names (Continent and Temperature) have been changed to Region and Climate respectively.**   | | | Table 1 | |
|  | | In Table 2, consider adding a range in the fourth column for completeness | **The word “range” was added in the fourth column of Table 2 as advised. “Temperature range for IHS reproduction”** Climate \| **IHS species** \| **Temperature range for IHS growth** \| **Temperature range for IHS reproduction** \| **Temperature range for IHS survival** \| Temperature range for the development of *Fasciola* spp intramolluscan stages | | | Table 2 | |
|  | | Although *Radix sp* has been cited in Table 1 and in the text, it is not clear how this data is related in both tables. | **We acknowledge this comment. The authors agreed to use the term *Radix natalensis* for all species written using the old name *Lymnaea natalensis* to avoid confusion and to maintain consistency in the revised manuscript. Therefore the data on *Lymnaea natalensis* was treated as the data for *Radix natalensis.*** | | | Table 1 and Table 2 | |
|  | | To enhance reader comprehension, the authors should avoid inserting paper codes in the middle of sentences. | **The issue of inserting paper codes in the middle of sentences was addressed in the revised manuscript.** | |  | | |
|  | | An English review is necessary to refine the manuscript's elegance. | **We revised the manuscript paying attention to grammatical issues.** | | |  | |
|  | | The inclusion of the mathematical modelling section in the results part requires clarification. It would be helpful to mention this in the Materials and Methods section and provide a summary of the most relevant models and outcomes | **The raised query was addressed by expanding the search for mathematical model studies on the effect of temperature on fascioliasis transmission. The following search terms were used: “Mathematical Models AND Temperature AND Fascioliasis AND Intermediate Hosts”. The following articles were selected: Hope-Cawdery M. Changing temperatures and prediction models for the liver fluke (*Fasciola hepatica*). Journal of Thermal Biology. 1981;6(4):403-8., Rapsch C, Dahinden T, Heinzmann D, Torgerson PR, Braun U, Deplazes P, et al. An interactive map to assess the potential spread of *Galba truncatula* and the free-living stages of *Fasciola hepatica* in Switzerland. Veterinary Parasitology. 2008;154(3-4):242-9.**  **Summary: “ The model by Rapsch et al. [2] suggested that the risk for fascioliasis is high between the temperature range of 10-25°C and low at temperatures below and above that range. Another model by Hope-Cawdery [3] predicted that the survival of extra-mammalian stages of liver fluke occurs optimally at a temperature range of 10-30°C”.** | | | Line 118  Lines 228-233 | |
| **Overall comments** | | | | | | | |
|  | | Overall, this is a well-conducted study that provides valuable insights into temperature-dependent stages and the biology of IH snails and Fasciola spp. However, the results section lacks summarization of each presented topic. Moreover, the discussion section is challenging to follow, requiring a complete reorganization to clarify the influences of temperature ranges on IH snail biology (linking aspects such as survival, life history, growth, and reproduction). I recommend addressing these issues before proceeding with further consideration for publication. | **We have made significant effort to make the discussion section less challenging to follow. In this regard we added summary statements at the end of each subtopic on the effect of temperature on growth, fecundity, and survival of *Fasciola* intermediate hosts as highlighted in the revised results sections and the reorganization of the discussion sections was done by discussing the actual results observed about the influence of temperature on life history traits of intermediate host snails fascioliasis and on the development of intramolluscan larval stages of the trematodes. Statements were moved to align with the issues discussed and other statements were removed from the discussion section completely*.*** | | | Results and Discussion sections. | |
| **Independent Review Report, Reviewer 2** | | | | | | | |
| **Abstract** | | | | | | | |
| Line 26  Lines 36-38 | | Line 26 – you say Intermediate Host Snails (IHS) – Should maintain the same criteria during the manuscript (Ex: Lines 36,37 and 38) | **We acknowledge this comment. The issue of maintaining the term Intermediate Host Snails (IHS) was resolved in the revised manuscript.** | | | Line 29, line 42 line 42 and line 47 | |
| **Background** | | | | | | | |
| Line 66 | | Liver fluke is the parasite, not the disease. | **The term liver fluke was replaced by the terms liver rot and distomatosis in the revised manuscript.** | | | Lines 72 | |
| Line 71 | | Sporocysts, rediae (not radiae) and cercariae are intramolluscan stages of trematodes, not intramolluscan tematodes | **The spelling for rediae was corrected in the revised manuscript.**  **The term intramolluscan stages of trematodes was used in the revised manuscript.**  **(Both *F. hepatica* and *F. gigantica* have a five-stage life cycle that includes an egg, miracidium, intramolluscan stages of trematodes (sporocysts, rediae, and cercariae), metacercariae, and adults).** | | | Lines 77-78 | |
| Line 75 | | *Galba truncatula* (also called *Lymnaea truncatula*). | ***The statement Galba truncatula (Lymnaea truncatula)* was rewritten as *Galba truncatula* (also called *Lymnaea truncatula)* as suggested*. “Galba truncatula* (also called *Lymnaea truncatula*) is involved in the transmission *of F. hepatica”.*** | | | Line 82-84 | |
| Line 75 | | Temperate regions. | *The statement Galba truncatula* (also called *Lymnaea truncatula*) is involved in the transmission of *F. hepatica* in the temperate was rewritten as: ***Galba truncatula* (also called *Lymnaea truncatula*) is involved in the transmission of *F. hepatica* in the temperate regions, while *Radix natalensis* ( also called *Lymnaea natalensis*) is involved in the transmission of *F. gigantica* in the tropical regions [4, 5].** | | | Lines 83 | |
| Line 76 | | Galba natalensis (also called *Lymnaea natalensis*) – not *Lymnaea truncatula*. | **We acknowelege this comment*.* The typo error *Galba natalensis* was supposed to be written as *Radix natalensis* and the statement was corrected to*:***  ***“Galba truncatula (also called Lymnaea truncatula)* is involved in the transmission of *F. hepatica* in the temperate regions, while *Radix natalensis (* also called *Lymnaea natalensis) is* involved in the transmission of *F. gigantica* in the tropics *[4, 5]”.*** | | | Lines 82-84 | |
| Line 75 | | Pseudosuccinea columella (also called Lymnaea columella). | ***Pseudosuccinea columella* (also called *Lymnaea columella*), an invasive species, has become an important intermediate host of both *Fasciola* species in many regions of the world [6].** | | | Lines 84-85 | |
| Line 88 | | Presence of the vector snails and parasites. | **The sentence was corrected to include the terms (presence of the vector snail) in the revised manuscript. “The transmission of fascioliasis is affected by several abiotic and biotic factors****, ranging from climatic factors such as temperature and rainfall to snail food, mammalian hosts, the latitude or altitude of the snail habitat, and the presence of vector snails and parasites [7, 8]”** | | | Lines 94-96 | |
| **Material and Methods** | | | | | | | |
| Line 117 | | You should explain why the search by Lymnaea, Galba, Pseudosuccinea and Radix. Maybe you should also search for *Fossaria* | ***We acknowledge this comment:* The main reason was to avoid missing any articles on the effect of temperature on the lymanaeid snails (IHS), since in the Lymnaea was used as the genus for the now *Galba/Fossaria,*** ***Austropeplea, Radix* and *Pseudosuccinea.* Also, the authors included the term *Fossaria* as one of the search terms*: “*Fasciolosis AND Temperature*, Lymnaea* OR *Radix* OR *Galba* OR** ***Austropeplea* OR *Pseudosuccinea* OR *Fossaria* AND *growth, fecundity, AND survival” as suggested.*** | | | Lines 113-115 | |
| Line 118 | | *Galba, Radix* (italics) | **We have italicized the words *Galba* and *Radix* in the revised manuscript.** | | | Lines 116-117 | |
| Line 126 | | *Fasciola hepatica* (italics) | ***Fasciola hepatica* has been Italicized in the revised manuscript*.*** | | | Lines 126 | |
| Line 131 | | If you say Lymnaea sp, must say Galba sp and Pseudosuccinea sp | **We correctected the comment raised as suggested*.* “Studies that do not include *Lymnaea* sp., *Fossaria sp*., *Galba sp*., *Radix sp.,* or *Pseudosuccinea sp”.*** | | | Line 131-132 | |
| Table1 | | Revise the italics; inoculated miracidia??? (page 12); Sometimes you say “x - yºC”, other "xºC - yºC" (should use the same criteria); | **We italicized all scientific names (genus and species names) in the revised manuscript.**  **The statement (Age, not the quantity of inoculated miracidia, significantly affected the**  **prepatent duration of *F. gigantica* inside its snail host, which was inversely related to temperature) was rewritten as “ The age of snails, not the number of miracidia to which they were exposed to, significantly affected the prepatent duration of *F. gigantica* inside its snail host, which was inversely related to temperature”.**  **We used the criterion “x-yºC” for temperature range in the revised manuscript.** | | | Table 1. Page 11. | |
| Line 175 | | Temperature is known to "have" impact "on" IHS growth | **We revised the statement as follows: “Temperature has been shown to affect the growth of IHS”** | | | Line 178 | |
| Line 212 | | Studies Stensgaard et al (???????) | **The statement was revised to:** “**Studies by Stensgaard et al. [9] and Pedersen et al. [10] have combined aspects of temperature and Geographic Information Systems (GIS) to predict the spatial distribution of the disease”.** | | | Line 222 | |
| *Line 225* | | snail intermediate hosts – should be SIH | **Snail intermediate hosts was replaced by (IHS) in the revised manuscript.** | | | Line 241 | |
| Lines 242, 247 | | sometimes is Lymnaeid, other lymnaeid (it is not the only case) | **The use of the term lymnaeid snails in lower cases was maintained in the revised manuscript.** | | | Lines 258, 261 | |
| Line 341 | | intramolluscan stages of *Fasciola hepatica* | **We revised the sub-heading sentence to: Temperature effect on the development of intramolluscan stages of Fasciola spp** | | | Line 340 | |
| Line 374 | | parasitization | **The subheading was revised to (Diseases Models) and the word (parasitisation) was removed.** | | | Line 368 | |
| Line 421 | | GIS (not a Geographic Information System (GIS)) | **We have maintained the use of “GIS” as the abbreviation for Geographic information System. This is supported in Literature by: Malone J, Gommes R, Hansen J, Yilma J, Slingenberg J, Snijders F, et al. A geographic information system on the potential distribution and abundance of *Fasciola hepatica* and *F. gigantica* in east Africa based on Food and Agriculture Organization databases. Veterinary Parasitology. 1998;78(2):87-101;** **Caminade, C., et al., Modelling recent and future climatic suitability for fasciolosis in Europe. Geospatial health, 2015. 9(2): p. 301-308 and** **Stensgaard A-S, Kabatereine N, Rahbek C, Kristensen T. Modeling freshwater snail habitat suitability and areas of potential snail-borne disease transmission in Uganda. Geospatial Health. 2006;1(1):93-104.** | | | Line 386  Line 442 | |
| **References** | | | | | | | |
| Line 500 | [5] incomplete | | | **The article was removed since it does not have a DOI and it’s a Book chapter by ITECHOPEN.** | | | Line 497 |
| Line 502 | [6] Tidman, R., et al… … (must see the recommended referencing style) You have much more cases like this. | | | **The query was addressed by using Endnote 20 Plos Medicine or Vancouver reference style.** | | | Line 497 |
| Line 516 | [11] incomplete | | | **We have completed that by adding “Mas-Coma S, Valero MA, Bargues MD. Fascioliasis. Digenetic Trematodes.2019:71-103. Adv Exp Med.2019:1154:71-103. doi: 10.1007/978-3-030-18616-6_4.”** | | | Line 510 |
| Line 537 | [20] incomplete | | | **We have completed that as follows: “Dillon RT.The ecology of freshwater molluscs.2 000.doi.org/10.1017/CBO9780511542008.”** | | | Line 539 |
| Line 540 | [22] incomplete | | | **We have completed that as follows: “Vázquez AA, Alda P, Lounnas M, Sabourin E, Alba A, Pointier J-P, et al. Lymnaeid snails hosts of Fasciola hepatica and Fasciola gigantica. 2018. doi.org/10.1079/PAVSNNR201813062.”** | | | Line 552 |
| Line 657 | [67] incomplete | | | **We have completed that as follows: “Vaughn CM. Effects of temperature on hatching and growth of Lymnaea stagnails appressa Say. American Midland Naturalist. 1953; 49,(1):214-228. doi.org/10.2307/2422289.”** | | | Line 656 |
| Line 702 | [86] “Note: MDPI stays neutral with regard to jurisdictional claims in published …” (????) | | | **Periago M, Valero M, Artigas P, Agramunt H, Bargues M, Curtale F, et al. Very high fascioliasis intensities in schoolchildren from Nile Delta Governorates, Egypt: The Old World highest burdens found in lowlands. Pathogens. 2021; 10: 1210. doi.org/10.3390/pathogens10091210** | | | Line 712 |
| **General comments** | | | | | | | |
|  | **General comments** | | | **The authors attended to all the issues raised.** | | |  |

References

1.	Stuen S, Ersdal C. Fasciolosis-An Increasing Challenge in the Sheep Industry. Animals (Basel). 2022;12(12):1491. Epub 20220608. doi: 10.3390/ani12121491. PubMed PMID: 35739828; PubMed Central PMCID: PMCPMC9219500.

2.	Rapsch C, Dahinden T, Heinzmann D, Torgerson PR, Braun U, Deplazes P, et al. An interactive map to assess the potential spread of *Lymnaea truncatula* and the free-living stages of *Fasciola hepatica* in Switzerland. Vet Parasitol. 2008;154(3-4):242-9. Epub 20080410. doi: 10.1016/j.vetpar.2008.03.030. PubMed PMID: 18495343.

3.	Hope-Cawdery M. Changing temperatures and prediction models for the liver fluke (	*Fasciola hepatica*). Journal of Thermal Biology. 1981;6(4):403-8.

4.	Mas-Coma S. Human fascioliasis: epidemiological patterns in human endemic areas of South America, Africa and Asia. Southeast Asian J Trop Med Public Health. 2004;35(Suppl 1):1-11.

5.	Mahulu A, Clewing C, Stelbrink B, Chibwana FD, Tumwebaze I, Russell Stothard J, et al. Cryptic intermediate snail host of the liver fluke *Fasciola hepatica* in Africa. Parasit Vectors. 2019;12(1):573. Epub 20191204. doi: 10.1186/s13071-019-3825-9. PubMed PMID: 31801595; PubMed Central PMCID: PMCPMC6894237.

6.	Ngcamphalala P, Malatji M, Mukaratirwa S. Geography and ecology of invasive *Pseudosuccinea columella* (Gastropoda: Lymnaeidae) and implications in the transmission of Fasciola species (Digenea: Fasciolidae)–a review. Journal of Helminthology. 2022;96:e1.

7.	Olkeba BK, Boets P, Mereta ST, Yeshigeta M, Akessa GM, Ambelu A, et al. Environmental and biotic factors affecting freshwater snail intermediate hosts in the Ethiopian Rift Valley region. Parasit Vectors. 2020;13(1):292. Epub 20200608. doi: 10.1186/s13071-020-04163-6. PubMed PMID: 32513295; PubMed Central PMCID: PMCPMC7282061.

8.	Fox NJ, White PC, McClean CJ, Marion G, Evans A, Hutchings MR. Predicting impacts of climate change on *Fasciola hepatica* risk. PLoS One. 2011;6(1):e16126. Epub 20110110. doi: 10.1371/journal.pone.0016126. PubMed PMID: 21249228; PubMed Central PMCID: PMCPMC3018428.

9.	Stensgaard AS, Jorgensen A, Kabatereine NB, Rahbek C, Kristensen TK. Modeling freshwater snail habitat suitability and areas of potential snail-borne disease transmission in Uganda. Geospat Health. 2006;1(1):93-104. doi: 10.4081/gh.2006.284. PubMed PMID: 18686235.

10.	Pedersen UB, Midzi N, Mduluza T, Soko W, Stensgaard AS, Vennervald BJ, et al. Modelling spatial distribution of snails transmitting parasitic worms with importance to human and animal health and analysis of distributional changes in relation to climate. Geospat Health. 2014;8(2):335-43. doi: 10.4081/gh.2014.23. PubMed PMID: 24893011.
